# Comparison of tissue processing methods for microvascular visualization in axolotls

**DOI:** 10.1016/j.mex.2017.08.001

**Published:** 2017-08-30

**Authors:** Rodrigo Montoro, Renee Dickie

**Affiliations:** Department of Biological Sciences, Towson University, 8000 York Road, Towson, MD 21252, USA

**Keywords:** Microvascular visualization in axolotls, Axolotl, Capillary, Regeneration, Whole mount, Salamander, Microscopy, Microvessels, Angiogenesis, Perfusion

## Abstract

The vascular system, the pipeline for oxygen and nutrient delivery to tissues, is essential for vertebrate development, growth, injury repair, and regeneration. With their capacity to regenerate entire appendages throughout their lifespan, axolotls are an unparalleled model for vertebrate regeneration, but they lack many of the molecular tools that facilitate vascular imaging in other animal models. The determination of vascular metrics requires high quality image data for the discrimination of vessels from background tissue. Quantification of the vasculature using perfused, cleared specimens is well-established in mammalian systems, but has not been widely employed in amphibians. The objective of this study was to optimize tissue preparation methods for the visualization of the microvascular network in axolotls, providing a basis for the quantification of regenerative angiogenesis. To accomplish this aim, we performed intracardiac perfusion of pigment-based contrast agents and evaluated aqueous and non-aqueous clearing techniques. The methods were verified by comparing the quality of the vascular images and the observable vascular density across treatment groups. Simple and inexpensive, these tissue processing techniques will be of use in studies assessing vascular growth and remodeling within the context of regeneration. Advantages of this method include:

• Higher contrast of the vasculature within the 3D context of the surrounding tissue

• Enhanced detection of microvasculature facilitating vascular quantification

• Compatibility with other labeling techniques

## Graphical abstract

**Figure:**
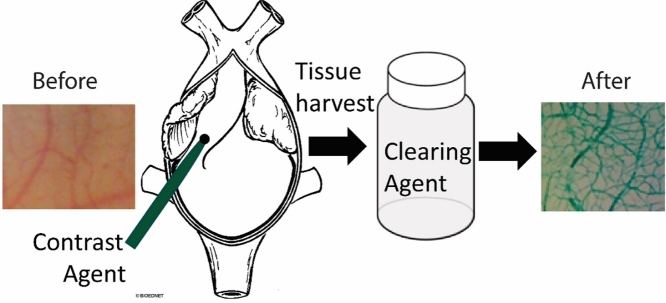

## Method details

### Rationale

Characterization of the vasculature is essential to understanding the complex role that blood vessels play during regeneration [1], [2]. Well defined vessels, distinct from background tissue, are necessary for the quantification of vascular image data. In other regenerating vertebrate models systems, such as zebrafish, transgenic lines with fluorescently labelled vascular endothelial cells greatly facilitate vessel analysis [3]. Techniques for vascular visualization in salamanders, however, are still in their infancy.

In mammalian models, procedures for ink microangiography and optical clearing to visualize the vasculature in both healthy and tumor tissues [4], [5], [6], [7], [8], [9], [10], [11], [12], [13], [14], [15] are well refined. The technique is sensitive; perfusion-based methods can result in a greater number of labelled vessels than immunohistochemical labeling [7]. While quantification of perfused vessels has been widely employed to examine mammalian vasculature and angiogenesis (e.g., [15], [16], [17], [18], [19], [20]), we are not aware of any literature applying these techniques to allow quantification of urodele angiogenesis. Here, we use an alternative contrast agent, green pigment based ink, and compare aqueous and nonaqueous optical clearing methods, towards the goal of optimizing vascular image quality and facilitating quantification of the regenerative vasculature. The technique was assessed by comparing the clarity of the vascular network and the detectable vascular density across the treatment groups. The whole mount procedures described below provide a flexible system that preserves the three dimensional architecture of the vasculature and allows for more accurate estimation of the areal density of the intact and regenerating microvessels.

## Methods

Using the methods of [21], [22] as a starting point, we describe modified and expanded procedures for the perfusion of the vascular tree of axolotls, followed by tissue clearing techniques to reduce light scattering during microscopy.

### Animal maintenance

Salamander maintenance followed standard procedures [23], [24], outlined here briefly. Juvenile axolotls (*Ambystoma mexicanum*, albino strain) were purchased from the *Ambystoma* Genetic Stock Center (AGSC, Lexington, KY) and allowed to acclimate for at least a month prior to any experiments. The animals were housed individually in plastic tanks within an 18–20 °C room with natural light. Tank solution consisted 40% Holtfreter’s salts [24] in dechlorinated water, manually changed on alternate days. The animals were fed every other day with anchovy-based food pellets (Rangen, Buhl, ID). All animal procedures were in accordance with an approved Institutional Animal Care and Use protocol and all local, state, and federal guidelines.

### Tail amputations

To assess regenerated vasculature, the tail tips of adult (∼12 cm snout vent length) axolotls were amputated and then allowed to regrow. Animals were first anesthetized until they were no longer responsive by immersion in 0.03% (wt./vol.) benzocaine (Sigma-Aldrich) diluted in 40% Holtfreter’s solution. A methanol cleaned scalpel was then used to amputate the distal ∼25% of the tail length. The animals were then returned to individual tanks containing fresh Holtfreter’s solution. The salamanders were allowed to regenerate their tails for four weeks prior to vascular perfusion and imaging.

### Intracardiac vascular perfusion with contrast agent

Axolotls with either intact tails or tails that had been allowed to regenerate following amputation (n = 5) were used in these experiments. Animals were euthanized by anesthetic overdose via prolonged immersion in 0.1% benzocaine (Sigma-Aldrich, St. Louis, MO) diluted in 40% Holtfreter’s solution. Ten minutes after complete loss of reactivity (unresponsive to toe pinch, unable to right itself), the animal was removed from the benzocaine solution. The tail tip was then digitally imaged under a Bausch and Lomb stereomicroscope to document the vasculature prior to the perfusion with contrast agent.

Perfusion procedures were modified from [21], [22]. The animal was pinned ventral side up on a dissection tray. Heparin was not administered prior to perfusion since no clotting was observed during the procedure. The abdomino-thoracic cavity was opened as follows. A scalpel was used to first make a shallow (just through the integument and body wall) ∼3 cm longitudinal incision just off the midline and posterior to the pectoral girdle. This was followed by a shallow transverse incision at the anterior end of the longitudinal incision. To ensure that no large vessels were severed or organs were nicked as the heart was exposed, we placed a blunt end probe through the incision opening and gently slid it rostrally, just deep to the body wall. Blunt dissecting scissors were then used to cut above the inserted probe, extending the incision towards the throat, and then transversely. The resultant body flaps were reflected and pinned back to clearly expose the heart.

**Fig. 1:**
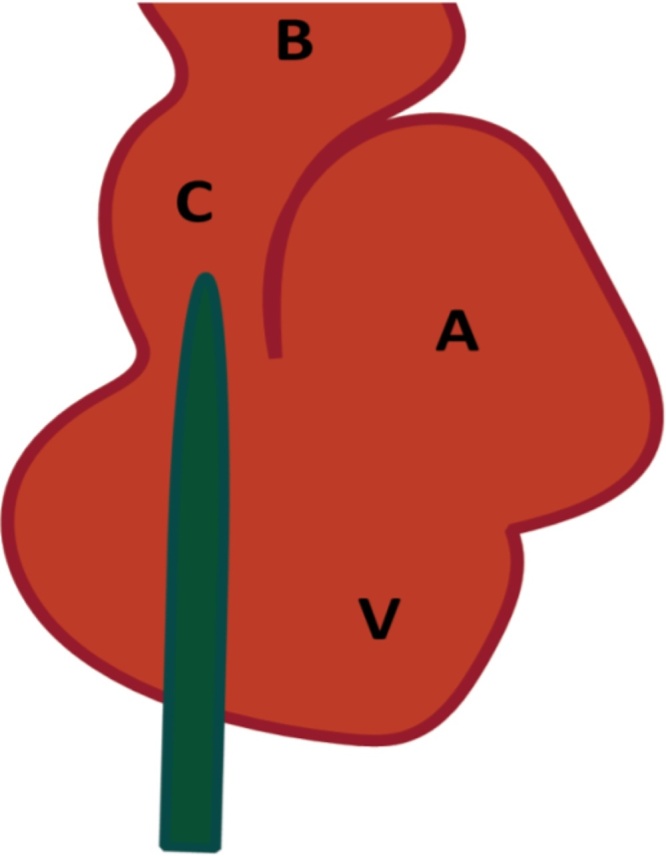
Stylized ventral view of heart showing positioning of the perfusion needle through the ventricle into the conus. Bulbus arteriosus (B), conus arteriosus (C), ventricle (V), atrium (A). Sinus venosus is dorsal.

A 25 gauge ball tipped needle or dulled 25 g syringe needle was attached to a 5 ml syringe containing 4 ml of a pigment suspension, either the more commonly employed colloidal carbon black or green pigment-based ink (Higgins, Chartpak, Leeds, MA). The needle was primed to avoid air bubbles, by holding the syringe upwards while depressing the plunger slightly until a drop of pigment emerged from the needle. A scalpel was used to make a tiny incision through the cardiac muscle at the apex of the ventricle. The needle was then quickly and gently inserted into the ventricle and just into the conus arteriosus (Fig. 1). A vascular clamp, small hemostat, or suture thread can be used to secure the needle within the heart and prevent blood leakage. The sinus venosus was nicked to allow blood and perfusate outflow. The syringe plunger was depressed very slowly and steadily to perfuse the microvasculature over a period of at least 2 min to allow capillary filling. Alternatively, perfusion can be controlled by placing the syringe in an infusion pump or using a gravity based system (100 cm above heart corresponding to 74 mmHg). Perfusion was verified under a stereomicroscope by observation of blanching of the tissue and the vessels changing color as they filled with the pigment.

### Tissue harvesting and fixation

All reagents were purchased from Fisher Scientific (Waltham, MA) unless otherwise noted. Once perfusion was complete, the needle was removed from the heart and the entire specimen was refrigerated briefly ( > 30 min) prior to tissue harvest. Because our lab is interested in caudal regeneration, we harvested the distal tail region, but since the perfusion is systemic, the technique is suitable for many other tissues of interest as well. The samples were fixed by immersion in 4% paraformaldehyde, pH 7.4, for 24 h at 4 °C. Perfusion fixation did not appear to be necessary to preserve morphology, but if larger organs were of interest, the vasculature could be perfused with fixative prior to contrast agent perfusion.

Chemical bleaching of tissue specimens helps remove endogenous pigment that absorbs light. The pale skinned albino and white strain axolotls did not require depigmenting. The darker wild type strain benefits from depigmentation; this can be done by submerging the tissue sample in a 3% solution of hydrogen peroxide until the tissue is bleached, as described in [25].

### Optical tissue clearing

Unlike the other classic vertebrate model for regeneration, larval zebrafish, which are more transparent, axolotl tissue is opaque, causing excessive light scattering during microscopic examination. In order to render the tissue optically clear for vascular imaging, tissue specimens were run through an ascending series of clearing agent. We compared the effects of three clearing agents on vessel visualization: aqueous clearing using glycerol, and dehydration-based clearing using BABB (benzyl alcohol benzyl benzoate) or methyl salicylate. Clearing procedures were adapted from [8], [26], [27].

Specimens were washed extensively prior to clearing to remove the paraformaldehyde fixative. For tissue processing, the tissue was cut into smaller samples, sized approximately 1 × 1 × 0.5 cm. The times for the clearing steps given below are minimal times; longer times are necessary for larger tissue samples. Each step was performed using an excess (>5 ml) of reagent.

### Aqueous clearing

This technique reduces light scattering through immersion in glycerol, a high refractive index solution. Washed, fixed tissue was run through an ascending glycerol series (3:1, 1:1, and 1:3 solutions of 0.5% KOH in water to glycerol) and followed by 100% glycerol, each step overnight or until tissue sinks, to ensure penetration. Specimens were stored and mounted for imaging in fresh 100% glycerol.

### Non-aqueous clearing

In this method of clearing, solvent-based dehydration to remove water and lipid is followed by refractive index matching using either BABB or methyl salicylate.

### BABB clearing

This clearing agent consists of a 1:2 mixture of benzyl alcohol to benzyl benzoate (BABB). For BABB clearing, fixed specimens were first washed and then dehydrated through a graded methanol series (50%, 75%, 95%, vol./vol. in water, >1 h per wash) to two washes of 100% methanol (overnight). Tissue was transferred to 100% BABB until the tissue sank and became transparent (methods adapted from Ref. [28]). An ascending BABB series (3:1, 1:1, 1:3, vol./vol. ethanol to BABB) can be used for delicate specimens. The samples were stored and later imaged in 100% BABB. BABB can dissolve certain plastics so the BABB series should be run using glassware; we used 20 ml glass scintillation vials. The clearing process renders the specimens both transparent and delicate, so care should be taken in handling.

### Methyl salicylate clearing

Washed, fixed tissue was dehydrated through a graded ethanol series and then immersed in methyl salicylate overnight or until fully transparent. Specimens were stored and imaged in 100% methyl salicylate.

### Imaging

Cleared specimens were digitally imaged in whole mount using a stereomicroscope for low magnification shots capturing the entire tail tip, or using a Nikon Labophot 2 compound microscope for higher magnification images. To prevent clearing agent from damaging compound microscopes, specimens can be examined within imaging chambers filled with clearing agent, coverslipped, and sealed with clear nail polish. Images were captured under the same settings for light, orientation, etc. in order to allow comparison of treatment groups. In some cases, images were cropped for presentation but there was no additional post-processing image manipulation. Because these are thick specimens with vessels located at various depths within the tissue, it is not always possible to keep all vessels fully in focus within a single image using routine light microscopy. If this is an issue, confocal microscopy can be used to collect 3D image stacks through the tissue [8].

### Vascular quantification

To help validate that the tissue processing procedures result in superior vascular images, the following image analysis was performed. To compare the amount of vasculature that was visible in unprocessed tissue, ink-perfused tissue, and ink-perfused tissue cleared by different methods, the vascular areal density was calculated for each group. Image processing and quantification were performed using ImageJ software [29]. To distinguish between vessels and background, raw digital images were segmented by manual thresholding of pixel intensity. In each randomly selected region of interest, the area occupied by vessels was divided by the area occupied by tissue to calculate vascular areal density. The total number of fields of view quantified was ∼70. Results are reported as means ±95% confidence interval.

## Method validation

**Fig. 2:**
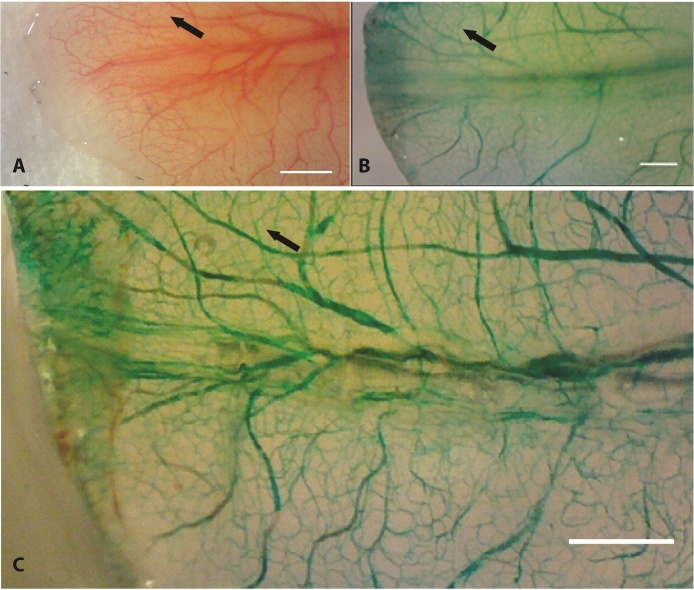
Perfusion with contrast agent followed by glycerol clearing of tissue improves contrast and visibility of vasculature compared to unprocessed tissue. A) *In vivo*, whole mount image of tail tip of an adult albino axolotl before perfusion with contrast agent. Vessels are red because they are filled with blood. B) The same salamander tail tip *ex vivo* after perfusion with contrast agent but prior to optical clearing. Vessels are green because the vasculature has been filled with green pigment-based ink. C) Perfused tail tip after optical tissue clearing with glycerol. Capillary networks are easier to visualize after perfusion with contrast agent and clearing (C) compared to unprocessed tissue (A). Arrows point to same region in tail. Scale bars = 1 mm.

**Fig. 3:**
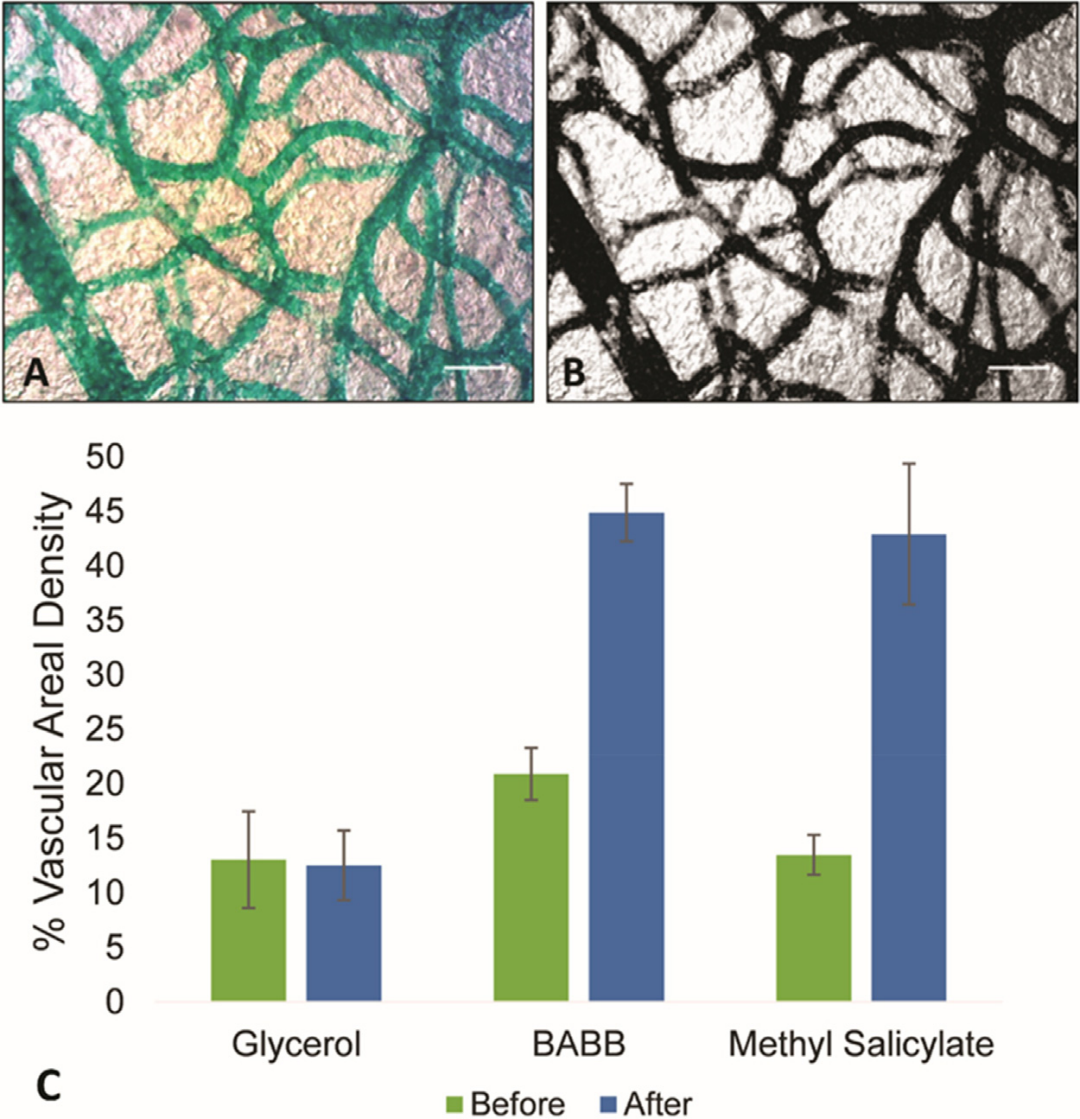
Nonaqueous clearing methods facilitated vascular quantification. Regions of interest (A) were segmented (B) for quantification of the vasculature. Scale bars = 100 μm. C) Plot compares detectable vasculature within the same tissue sample prior to tissue clearing and afterwards. The detectable vascular areal density was higher in BABB and methyl salicylate cleared tissue compared to uncleared tissue (*t*-test, p < 0.05). Error bars represent 95% CI.

We compared our ability to detect the vasculature in unprocessed tissue versus treated specimens. The circulating blood allows the visualization of many vessels *in vivo* without any additional tissue processing (Fig. 2A). Following intracardiac injection of ink, all animals displayed well-perfused vasculature. The vascular architecture of the tail was well defined, forming a mesh-like network. In order to validate that the technique produced superior visualization of the microvasculature compared to unprocessed tissue, we compared images of the microcirculation of the same animal before and after the perfusion and clearing procedures. In all cases, the perfused tissue produced superior images with higher contrast and clarity of the vasculature. Additional capillary networks that were not distinct in the unperfused tissue became clear in the tissue perfused with contrast agent and cleared (Fig. 2C). Higher magnification imaging to view the microvasculature confirmed a well perfused capillary network (Fig. 3A).

**Fig. 4:**
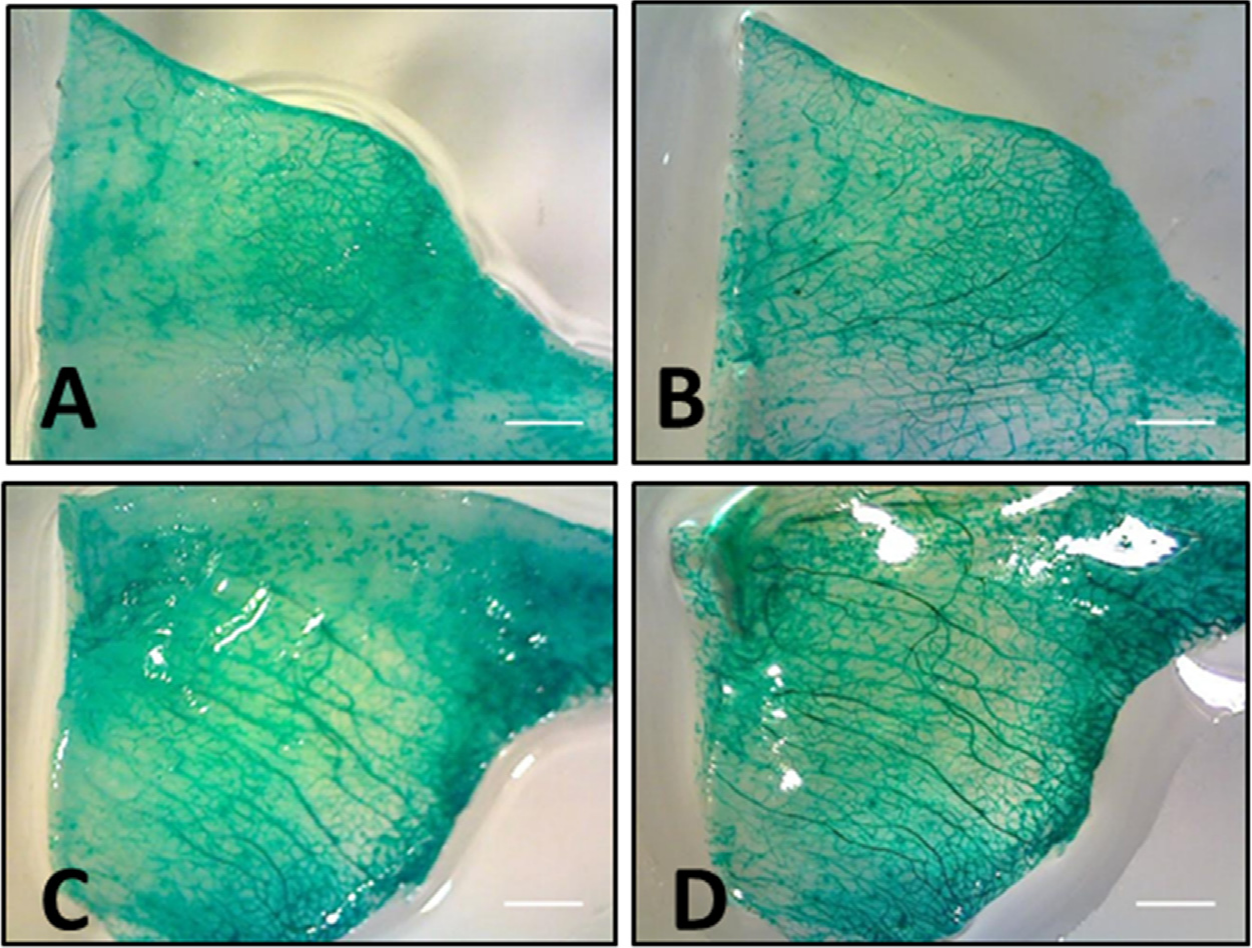
Nonaqueous clearing permits better visualization of the vasculature than uncleared tissue. An albino salamander was perfused with green pigment and samples of the regenerated tail were first imaged uncleared (A, C) and then after clearing with either methyl salicylate (B), or benzyl alcohol-benzyl benzoate (D). Scale bars = 1 mm.

While perfusion with contrast agent produces better vascular images than unperfused animals, the opacity of the surrounding tissue presents an obstacle to the detection of the entire vascular network. Clearing of the tissue was necessary to further enhance the visibility of the vessels (Fig. 4). Comparison of the same tissue sample before and after clearing indicated that all clearing methods (glycerol, BABB, methyl salicylate) produced visually better results than uncleared tissue. Glycerol clearing is the most rapid technique as no dehydration step is required. However, non-aqueous clearing, either with BABB or methyl salicylate produced superior results to aqueous clearing with glycerol. Both methyl salicylate and BABB clearing significantly increased the detectable areal vascular density (Fig. 3). This is consistent with the results for aqueous versus solvent based clearing found for other tissue types/animal models [30], [31]. This improved clarity may come at the cost, however, of some tissue shrinkage associated with non-aqueous methods [31]. Vessels are easiest to see in the albino, as opposed to wildtype, animals due to the absence of pigmentation. The clearest, most easily quantified vasculature was obtained using albino animals with green pigment contrast agent and methyl salicylate clearing.

## Additional information

### Advantages

Understanding the role of angiogenesis in regeneration is essential and requires the use of emerging model organisms [32], yet few specimen preparation techniques designed to enhance microvascular visualization in the best vertebrate regenerators, axolotls, have been described. Quantitative image analysis of vascular parameters such as vessel density and tortuosity require well-defined images of the microvasculature. Towards this goal, we adapted specimen preparation techniques for the visualization of the spatial distribution of the intact vascular bed and regenerating neovasculature.

Whole mount imaging has the advantage of allowing a three-dimensional analysis of the vasculature, without the need for time consuming paraffin processing and reconstruction from serial sections. Unlike vascular corrosion casting methods, which destroy surrounding tissues, the procedure described here allows analysis of the vasculature within the context of surrounding tissues. Ink based perfusion is usually performed with black colloidal carbon or India ink, but we found that other micropulverized pigment based inks (e.g., Higgins Pigment-based Drawing Ink Green 44695, blue 44685, and violet 44675) can also withstand the clearing process, providing flexibility in the color of the perfusion label that facilitates co-labeling with other markers. The clearing agents used here are known to be compatible with other staining methods. For example, glycerol is compatible with skeletal labeling methods [26], BABB is compatible with additional labelling via immunohistochemistry [28] and methyl salicylate preserves fluorescence [33]. Thus these techniques can be combined with immunohistochemical and other labels for simultaneous visualization of the vasculature with other structures [8], providing the foundation, for example, for co-labeling the microvasculature along with its associated pericytes for the assessment of vascular maturity. While we used standard light microscopy, clearing methods combined with vascular perfusion are well suited to other imaging modalities as well. For example, in other model organisms, ink angiography has been used with confocal imaging [8], [34] and Micro-Optical Sectioning Tomography [5]. The clear discrimination of vessels from tissue that this technique provides facilitates vessel segmentation and quantification of vascular metrics such as areal density and tortuosity [35].
